# *Klebsiella oxytoca* Endocarditis With Complete Heart Block

**DOI:** 10.1177/2324709616663232

**Published:** 2016-08-30

**Authors:** Saad Ullah, Omar Elbita, Mahmoud Abdelghany, Hassan Tahir, Puneet Tuli, Waseem Zaid Alkilani, Joshan Suri

**Affiliations:** 1 Comemaugh Memorial Medical Center, Johnstown, PA, USA; 2 SUNY Upstate Medical University, Syracuse, NY, USA

**Keywords:** *Klebsiella oxytoca*, endocarditis, heart block

## Abstract

Gram-negative bacterial endocarditis causes 5% of all bacterial endocarditis. Among gram-negative bacteria, *Klebsiella* species are rare causes of native valve endocarditis. *Klebsiella oxytoca* is an extremely rare subspecies that can infrequently cause endocarditis and is associated with poor outcome. We report a case of *Klebsiella oxytoca* endocarditis in an elderly man who initially presented with stroke but later developed sepsis and heart block secondary to endocarditis.

## Introduction

The incidence of infective endocarditis (IE) is rising. In the United States, every year, 10 000 to 15 000 new cases of endocarditis are reported. The delay in diagnosis contributes to the continuing high morbidity and mortality (20%) despite the advances in diagnostic tools.^1^ Conduction abnormality, in particular complete heart block, is an infrequent manifestation of IE that may require prompt ventricular pacing and cardiothoracic surgery intervention.^2^

## Case Presentation

**Figure 1.**
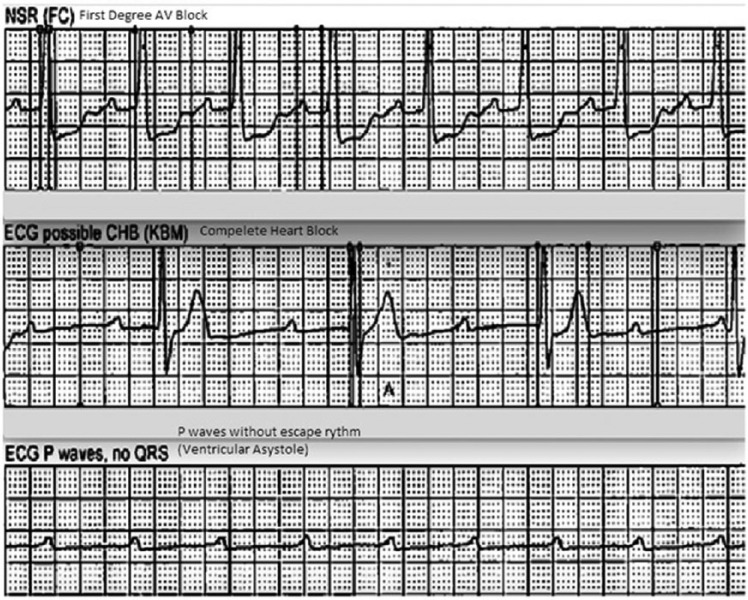
Strips showing varying degrees of heart blocks.

**Figure 2.**
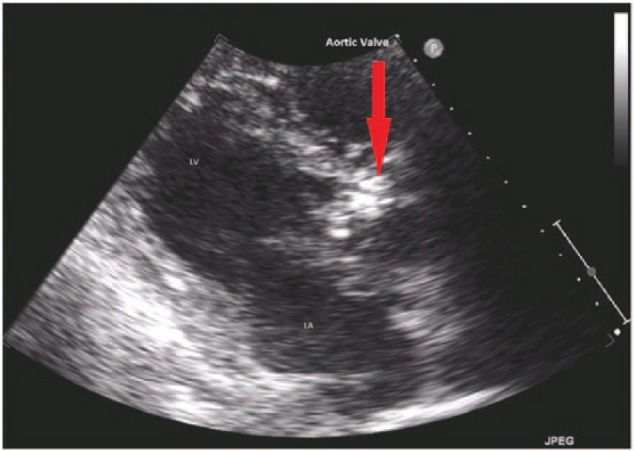
Aortic valve vegetation.

A 76-year-old Caucasian male was admitted to our hospital with acute onset of left-sided weakness. His past medical history included osteoarthritis only, and his surgical history included bilateral knee replacements. There was no history of cholecystitis, gallstones, pancreatitis, or abdominal surgeries in the past. He denied any fever, chills, shortness of breath, chest pain, palpitations, abdominal pain, nausea, vomiting, and constipation at the time of admission. He was an active smoker (1 pack of cigarettes daily) but denied use of alcohol or illicit drugs. On neurological exam, there was contralateral hemiplegia and hemiparesis of left face, arm, and leg. Power was 0/5 in left upper and lower extremity, and reflexes were absent bilaterally. His speech was slurred, and he failed swallowing evaluation in the emergency department. He was not a tissue plasminogen activator (TPA) candidate as the time of onset of stroke was unknown. Magnetic resonance imaging of brain without contrast showed subacute to acute infarct in the right middle cerebral artery territory. Magnetic resonance angiography of the brain showed acute occlusion of middle cerebral artery in the proximal M1 segment. Carotid Doppler showed mild stenosis of internal carotid arteries bilaterally. Transthoracic echocardiogram was done to rule out embolic source of stroke, which revealed ejection fraction of 45% with no valvular abnormalities and shunting. His chest X-ray was unremarkable. Two days later, he suffered septic shock with a fever of 39°C and hypotension. Blood, urine, and respiratory cultures were obtained, and broad-spectrum antibiotics (vancomycin and zosyn) were started empirically. Two blood cultures were positive for *Klebsiella oxytoca*, which was sensitive to ceftriaxone, levofloxacin, and gentamycin and resistant to ampicillin and cefazolin. Urine and respiratory cultures did not grow any bacteria. Antibiotics were tailored to ceftriaxone based on the sensitivity results. Computed tomography scan of the abdomen, as part of workup for septic shock, showed dilated common bile duct. Right upper quadrant ultrasound showed cholecystitis with clinical suspicion of acute cholangitis. He did have right upper quadrant pain, fever, and elevated alkaline phosphatase suggestive of acute cholangitis. He underwent percutaneous cholecystostomy and an endoscopic retrograde cholangiopancreatography. Drainage culture showed carbapenem-resistant *Escherichia coli*. He continued to require vasopressors and fluid resuscitation despite repeat negative blood cultures. On hospital day 12, he developed a new-onset intermittent complete heart block with significant pauses (Figure 1), requiring temporary transvenous pacing. Transthoracic echocardiography (TTE) detected severe aortic incompetence, with a large aortic vegetation measuring 1.7 × 0.6 cm (Figure 2). This was a new finding compared to a TTE done on the second day of admission. He did not have any chest pain, shortness of breath, cough, or palpitations. No signs of IE such as petechiae, splinter hemorrhages, Osler nodes, Roth spots, or Janeway lesions were appreciated on physical examination. The patient died before obtaining a transesophageal echocardiography in the process of evaluation for cardiac surgery. Autopsy was declined by the family.

The diagnosis of *Klebsiella* endocarditis was inferred from positive blood cultures, aortic valve vegetation on TTE, and new-onset heart block. The likely source of *Klebsiella* bacteremia was acute cholangitis and cholecystitis. The presenting symptom of stroke was possibly secondary to emboli from the endocarditis. The patient progressed despite timely, appropriate intravenous antibiotics to manifest heart block, which progressed to asystole.

## Discussion

*Klebsiella* species account for 1.2% of gram negative native valve endocarditis and 4.1% of prosthetic valve endocarditis and is associated with higher rate of complications and mortality than other gram-negative endocarditis.^3^ Historically, predisposing factors of IE were rheumatic heart disease or poor dental hygiene. Today, with an aging population, the higher prevalence of degenerative valvular disease, multiple comorbidities, drug abuse, and the use of intracardiac devices all contributed to the evolution in epidemiology and microbiology of IE including the rise in gram-negative IE cases.^2^ Gram-negative bacteria rarely infect native heart valves owing to less adherent potential to the endocardium in contrast to higher virulence organisms such as *Staphylococci*.^4^

Our patient had the host risk factors for developing gram-negative IE including age, gall bladder disease, prolonged hospital stay, and the gram-negative bacteremia. *Klebsiella oxytoca* bacteremia is commonly seen in the setting of hepatobiliary tract, urinary tract, skin and soft tissue, and peritoneal infection.^5^ Lin et al^6^ reported biliary tract infection in more than 50% of patients with *Klebsiella oxytoca* bacteremia; however, none of these cases were screened for IE. Although the gall bladder drainage did not grow *Klebsiella oxytoca*, we believe that the port of entry in our patient was the gall bladder infection.

Diagnosis of gram-negative IE in our patient was based on modified Duke’s criteria with 1 major (oscillating intracardiac mass on the aortic valve) and 3 minor criteria (aortic regurgitation, fever, and 2 positive blood cultures). The pathogenesis of heart block with IE is postulated to be from direct spread from the infected valve ring to the conduction system or micro-emboli through coronary arteries.^2^ Transesophageal echocardiography is usually required to confirm the diagnosis.

In our patient, the multiple comorbidities, the unusual organism, and the presence of cholecystitis led to the delay in diagnosis of IE. Persistent gram-negative bacteremia could have raised the suspicion for IE, but none of the subsequent blood cultures were positive in our patient. Our patient did not have any fever, chills, chest pain, and shortness of breath, which makes it unlikely that the patient had IE at the time of admission. Similarly, he denied any symptoms of cholecystitis in the emergency department. We believe cholecystitis and IE occurred during the hospital stay, the cause of which was unknown to us. Extensive workup was done to find the alternate source of gram-negative bacteremia. He did not have any central line or Foley’s catheter, and urine and sputum culture were negative. To our knowledge, this is the third reported case of *Klebsiella oxytoca* endocarditis in the English medical literature, and the first that led to death.

The presumptive diagnosis of *Klebsiella oxytoca* endocarditis was made based on positive blood cultures, heart block, and vegetation on TTE. The definite diagnosis would have required autopsy and biopsy of aortic valve, which unfortunately the patient’s family declined.

## References

[1] Slipczuk L Codolosa JN Davila CD Infective endocarditis epidemiology over five decades: a systematic review. PLoS One. 2013;8(12):e82665. doi:10.1371/journal.pone.0082665 24349331 PMC3857279

[2] Brown RE Chiaco JMC Dillon JL Catherwood E Ornvold K Infective endocarditis presenting as complete heart block with an unexpected finding of a cardiac abscess and purulent pericarditis. J Clin Med Res. 2015;7:890-895. doi:10.14740/jocmr2228w 26491503 PMC4596272

[3] Roodpeyma S *Klebsiella pneumoniae* endocarditis. Arch Pediatr Infect Dis 2015;3(2):e20079. doi:10.5812/pedinfect.20079

[4] Raza SS Sultan OW Sohail MR Gram-negative bacterial endocarditis in adults: state-of-the-heart. Expert Rev Anti Infect Ther. 2010;8:879-885. doi:10.1586/eri.10.76 20695743

[5] Chen JY Chen PS Chen YP Lee WT Lin LJ Community-acquired *Klebsiella oxytoca* endocarditis: a case report. J Infect. 2006;52(5):e129-e131. doi:10.1016/j.jinf.2005.08.015 16233915

[6] Lin RD Hsueh PR Chang SC Chen YC Hsieh WC Luh KT Bacteremia due to *Klebsiella oxytoca*: clinical features of patients and antimicrobial susceptibilities of the isolates. Clin Infect Dis. 1997;24:1217-1222. doi:10.1086/513637 9195086

